# First Real-Time Imaging of Acute Effects of Arteriovenous Fistula on Regional Distribution of Pulmonary Perfusion in a Novel Porcine Model

**DOI:** 10.33549/physiolres.935411

**Published:** 2025-02-01

**Authors:** João Batista BORGES, Anna VALERIANOVA, Leoš TEJKL, Jan MALIK, Mikuláš MLČEK, Otomar KITTNAR

**Affiliations:** 1 Institute of Physiology, The First Faculty of Medicine, Charles University, Prague, Czech Republic; 2 The Third Department of Internal Medicine, General University Hospital in Prague, The First Faculty of Medicine, Charles University, Prague, Czech Republic

**Keywords:** Arteriovenous fistula, Hyperkinetic circulation, Tissue perfusion, Animal model, Pulmonary blood flow

## Abstract

The effects of a large arteriovenous fistula (AVF) on pulmonary perfusion remains to be elucidated. We aimed to study, for the first time, the real-time acute effects of a large AVF on regional distribution of pulmonary perfusion in a novel porcine model. Ten healthy swine under general anesthesia were studied. AVF was created by the connection of femoral artery and femoral vein using high-diameter perfusion cannulas. The AVF was closed and after 30 min of stabilization the first values were recorded. The fistula was then opened, and new data were collected after reaching stable state. Continuous hemodynamic monitoring was performed throughout the protocol. The following functional images were analyzed by electrical impedance tomography (EIT): perfusion and ventilation distributions. We found an increased cardiac output and right ventricular work, which was strongly correlated to an increased pulmonary artery mean pressure (*r*=0.878, *P*=0.001). The ventral/dorsal ratio of pulmonary perfusion decreased from 1.9±1.0 to 1.5±0.7 (*P*=0.025). The percentage of total pulmonary blood flow through the dorsal lung region increased from 38.6±11.7 to 42.2±10.4 (*P*=0.016). In conclusion, we have used EIT for the first time for studying the acute effects of a large AVF on regional distribution of pulmonary perfusion in a novel porcine model. In this new experimental model of hyperkinetic circulation caused by AVF, we documented an increased percentage of total pulmonary blood flow through the dorsal lung region and a more homogeneous perfusion distribution.

## Introduction

An arteriovenous fistula (AVF) comprises a low-resistant circuit that bypass the resistant arteriolar beds [1]. AVFs can be engendered intentionally as a vascular access for hemodialysis [2], but can be also post-traumatic and congenital. An AVF leads to a substantial increase in arterial and venous flow and the vessels adapt to it by a complex of interesting processes. Some of these processes are specific to the particular organ circulation physiology and its interplay with others. A large AVF gives rise to a rapid decrease in systemic vascular resistance, increase in cardiac output (CO) and venous return, along with other hemodynamic effects. Some of these effects were previously described [3], but many of them were not yet fully studied.

The arterial tree in mammals contains high- and low-resistant beds. Low-resistant circuits guarantee quite stable blood supply into vital organs such as brain, kidneys, and coronary arteries. Because an AVF is also a low-resistant circuit, its concurrence with other low-resistant beds is an unfavorable possibility. For instance, clinical data pointed out a negative effect of an AVF in renal graft perfusion [4] and in cerebral oxygenation [5]. The possibility of developing coronary steal in patients having coronary artery bypass graft using internal thoracic artery and ipsilateral upper extremity arteriovenous hemodialysis fistula has been also reported [6,7]. Furthermore, it was associated with increased risk of cardiac events [8]. Very recently, our group reported, in a novel non-surgical porcine AVF model, that an increase in CO was linked with higher coronary blood flow, but at the cost of lower carotid perfusion and brain oxygenation [9].

The pulmonary vasculature is unparalleled in volume and function. During fetal life, the pulmonary circulation is typically a low flow, high-resistance circuit. During the transition to postnatal life, the pulmonary vasculature dilates, and becomes able to accommodate the entire CO, with high blood flow maintained at low intravascular pulmonary arterial pressure (PAP). When compared with the systemic circulation, pulmonary arteries have thinner walls with much less vascular smooth muscle and a relative lack of basal tone, probably a function of high production of endogenous vasodilators and low production of vasoconstrictors, resulting in a normal pulmonary vascular resistance, which is approximately one-tenth that of the systemic circulation. In the adult lung, factors controlling pulmonary blood flow include vascular structure, gravity, mechanical effects of breathing, and the influence of neural and humoral factors. However, the effects of AVF creation on regional distribution of pulmonary perfusion remains to be elucidated.

Electrical impedance tomography (EIT) is a noninvasive, radiation-free, real-time lung function imaging method [10]. Cyclic variations in pulmonary air and blood content are the major determinants for the changes in thoracic impedance. Besides features like being a bedside imaging tool and providing the possibility of around-the-clock monitoring, the high temporal resolution is a crucial aspect of EIT imaging that allows for the study not only of ventilation distribution [11,12], but also of faster physiological phenomena, such as pulmonary perfusion [13–18].

In the present study, we aimed to study, for the first time, the real-time acute effects of a large arteriovenous fistula on regional distribution of pulmonary perfusion in a novel porcine model.

## Methods

We performed a detailed new analysis of all the EIT perfusion and ventilation distributions data, as well as a detailed new analysis of all the continuous hemodynamic monitoring data, of a previously published experimental study in 11 pigs [9]. Here, 10 animals with complete EIT imaging and hemodynamic data were included.

The study protocol was approved by the Institutional Animal Care and Use Committee of the First Faculty of Medicine, Charles University (Ethical Committee License number MSMT-11198/2020-2). The study was performed in an accredited animal laboratory of the Institute of Physiology, First Faculty of Medicine, Charles University, in accordance with Act No. 246/1992 Coll., on the protection of animals against the cruelty that is harmonized with EU legislation. The “Principles of laboratory animal care” (NIH publication No. 86–23, revised 1985) were followed, as well as specific current version of the Czech Republic Law on the Protection of Animals.

### Investigational protocol

The experimental design has been previously described in detail [9]. Briefly, ten healthy swine were included into these new analyses. All animals were lying in the supine position and were under general anesthesia throughout the study. The mechanical ventilation settings were adjusted to maintain normal both oxygen and carbon dioxide levels within the arterial blood and remained unchanged onwards. AVF was produced by connecting the femoral artery and vein using high-diameter perfusion cannulas. The blood flow through the AVF was continuously measured as was also previously described in detail [9]. A proper clamp fixed around the arterial cannula made it possible to regulate the AVF blood flow and to close the fistula when necessary. After verifying an adequate AVF functioning, the AVF was closed by the clamp and after 30 min of stabilization the baseline measurements were collected (AVF_closed_). The fistula was then reopened and after animal stabilization new measurements were recorded (AVF_open_). AVF_open_ data represent measurements obtained after 60 min of opened AVF after reaching stable state.

### Hemodynamic measurements and computations

These hemodynamic measurements and computations have been previously described in detail [9]. Briefly, continuous invasive arterial blood pressure measurement was done using a catheter inserted in the contralateral femoral artery. Central venous pressure (CVP) was measured using a central venous catheter inserted in the left jugular artery. PAP, continuous measurement of mixed venous oximetry (SvO_2_), and CO measurements were collected using a Swan-Ganz catheter properly inserted in the pulmonary artery. Continuous electrocardiogram, peripheral oxygen saturation and end-tidal carbon dioxide monitoring were also implemented. Stroke volume (SV) was computed as SV = CO/heart rate (HR). The left ventricular stroke work was estimated as *SVx(MAP-PCW)×0.0136* (MAP = mean arterial pressure, PCW = pulmonary capillary wedge pressure). The right ventricular work was estimated as *SVx(PAMP-CVP)×0.0136* (PAMP = pulmonary artery mean pressure).

### EIT monitoring and measurements

Pulmonary EIT data were recorded at 50 Hz with 32 electrodes equidistantly placed around the circumference of the thorax just below the level of the axilla (Enlight, TIMPEL SA, São Paulo, Brazil) [15,16].

The following functional images were generated by EIT:

#### Pulmonary Perfusion distributions

They were obtained by injecting a bolus of 10 ml of a hypertonic solution (NaCl 10 %) into a central venous catheter during an expiratory breath hold for 20 s. Due to its high conductivity, NaCl 10 % acts as an EIT contrast agent [19], which after injection into the right atrium during apnea passes through the pulmonary circulation, thereby producing a dilution curve that follows typical first-pass kinetics. The resulting regional impedance curves are then analyzed to quantitatively assess regional perfusion [16,20,21], expressed as the percentage of total pulmonary blood flow through each of the regions-of-interest (ROIs), total 100 %.

#### Pulmonary Ventilation distributions

They were derived from relative impedance changes, which reliably track local, pixel-by-pixel changes in the content of air within the lung [11,22]. The *ventilation distributions* were expressed as the percentage of total pulmonary ventilation through each of the regions-of-interest (ROIs), total 100 %.

For the *ventilation distributions*, the following EIT-derived parameters were monitored and measured:

- Delta Z (ΔZ): variation of impedance during a tidal breath, both global (surrogate of V_T_) and regionally (surrogate of regional V_T_ distribution).
- Distribution of regional tidal ventilation was determined as the relation of regional ΔZ/total ΔZ, expressed in percentage, and was also used to estimated regional V_T_ (V_Tr_) = (regional ΔZ/total ΔZ) × total V_T_.

For these *Pulmonary Perfusion and Ventilation distributions* analyzes, the lungs were sub-segmented into the following ROIs: *Ventral* (upper lung or anterior half) and *Dorsal* (lower lung or posterior half).

The *Ventral/Dorsal* ratio of pulmonary perfusion was also calculated as the following: the percentage of total pulmonary blood flow through the *Ventral* ROI/the percentage of total pulmonary blood flow through the *Dorsal* ROI.

### Statistical analysis

Continuous variables are presented as mean ± SD. The Shapiro-Wilk test was used to test data for normality. The paired-samples *t*-test was used to determine whether the mean difference between paired observations is statistically significantly different from zero. Correlation analysis was performed according to Pearson. The statistical analyses were conducted with SPSS (version 25; IBM Corp, IBM SPSS Statistics for Windows, Armonk, NY). Individual *P* values to indicate statistical tests’ significance are reported where relevant. The *P*<0.05 was considered significant.

## Results

All the ten crossbred (Landrace × Large White) healthy female pigs (*Sus scrofa domestica*), 6–7 months old, and weighting 62.6±5.4 kg completed the whole study protocol and were included in these analyzes. All their data on hemodynamics, perfusion and ventilation distributions were available for analysis.

The AVF blood flow volume (Qa) was 2.14±0.54 l/min. It represented 24±4 % of baseline cardiac output.

### Hemodynamics

Immediately after AVF opening, the following changes of main hemodynamic parameters were observed: HR and PAP showed a continuous increase since AVF opening; MAP dropped rapidly, then rose after the initial dip, however, the resulting values remained lower than in baseline.

Hemodynamic changes after 60 min of opened AVF after reaching stable state are displayed in Table 1 (AVF_closed_ vs. AVF_open_). It was evidenced that an increased CO, and an increased right ventricular work that was strongly correlated to an increased PAMP (*r*=0.878, *P*=0.001). There was no significant correlation of right ventricular work with SV (*r*=0.256, *P*=0.48).

### Regional distribution of pulmonary perfusion

**Fig. 1:**
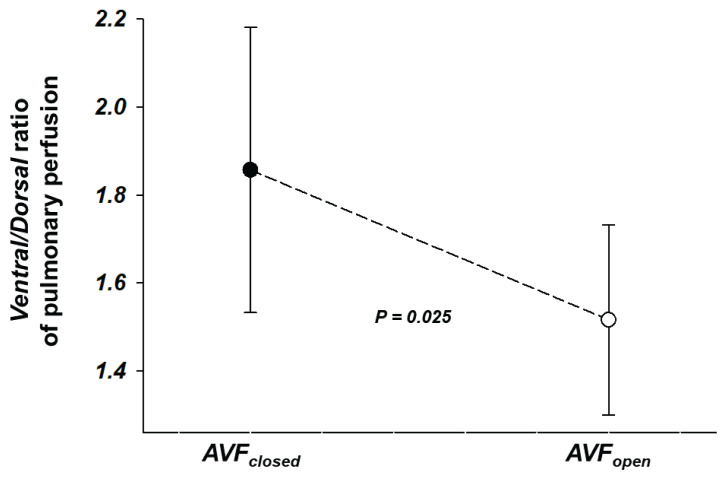
*Ventral/Dorsal* ratio of regional distribution of pulmonary perfusion by electrical impedance tomography. The lungs were sub-segmented into the following two regions: *Ventral* (upper lung or anterior half) and *Dorsal* (lower lung or posterior half). The *Ventral/Dorsal* ratio of pulmonary perfusion was calculated as the following: the percentage of total pulmonary blood flow through the *Ventral* region/the percentage of total pulmonary blood flow through the *Dorsal* region. Note that the *Ventral/Dorsal* ratio decreased significantly comparing closed arteriovenous fistula (AVF_closed_) vs. open arteriovenous fistula (AVF_open_). That points out a more homogeneous perfusion distribution after 60 min of opened AVF after reaching stable state (AVF_open_).

The *Ventral/Dorsal* ratio of pulmonary perfusion (Fig. 1) decreased significantly comparing AVF_closed_ vs. AVF_open_: 1.9±1.0 vs. 1.5±0.7 (*P*=0.025). That points out a more homogeneous perfusion distribution after 60 min of opened AVF after reaching stable state.

The percentage of total pulmonary blood flow through the *Dorsal* ROI increased significantly comparing AVF_closed_ vs. AVF_open_: 38.6±11.7 vs. 42.2±10.4 (*P*=0.016).

### Regional distribution of pulmonary ventilation

The percentage of total pulmonary ventilation through the *Dorsal* ROI, which did not change comparing AVF_closed_ vs. AVF_open_, was 30.4±14.6.

**Fig. 2:**
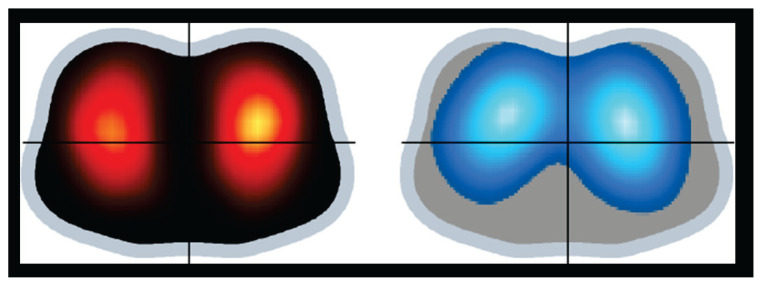
*Regional Distribution of Pulmonary Perfusion and Ventilation* by electrical impedance tomography – closed arteriovenous fistula. Representative EIT functional images illustrating the findings on regional distribution of pulmonary perfusion (red) and ventilation (blue) at closed arteriovenous fistula. Data were recorded after the arteriovenous fistula was closed and after 30 min of stabilization. Color scale of the EIT perfusion distribution image: the lighter the red the greater the regional perfusion. Color scale of the EIT ventilation distribution image: the lighter the blue the greater the regional ventilation.

**Fig. 3:**
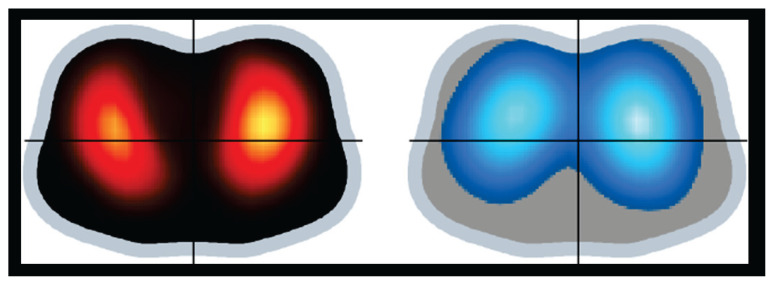
*Regional Distribution of Pulmonary Perfusion and Ventilation* by electrical impedance tomography – open arteriovenous fistula. Representative EIT functional images illustrating the findings on regional distribution of pulmonary perfusion (red) and ventilation (blue) at open arteriovenous fistula. Data were recorded after the fistula was reopened and after animal stabilization, after at least 1 h of observation (data represent measurements obtained after 60 min of opened AVF after reaching stable state). Color scale of the EIT perfusion distribution image: the lighter the red the greater the regional perfusion. Color scale of the EIT ventilation distribution image: the lighter the blue the greater the regional ventilation.

Figs 2 and 3 shows representative EIT functional images illustrating the findings in the distribution of pulmonary ventilation and perfusion at AVF_closed_ and AVF_open_.

## Discussion

We created a clinically relevant model of hyperkinetic circulation caused by a large arteriovenous fistula. And, by using EIT imaging, for the first time it was possible to study the real-time acute effects of such condition on regional distribution of pulmonary perfusion.

Both ventricles are exposed to volume overload with the presence of an AVF. But the right ventricle, in particular, also faces an increased pressure load, caused by increased pulmonary arterial pressure. Consequently, a significant increase in right ventricular work may arise. In a study with late-gestation fetal sheep, in which a right carotid artery-jugular vein fistula was surgically created [23], the authors documented the following: within 1 day of AVF creation, right ventricle output was 20 % higher in experimental than sham fetuses; right ventricle-to-body weight ratios were 35 % higher in the AVF group; and right ventricle cardiomyocytes grew longer in fetuses with an AVF. Indeed, cardiac hypertrophy is a well know consequence of AVF creation. Similar to Karamlou *et al*. findings, with the presence of an AVF we found increased cardiac output, stroke volume, pulmonary artery systolic and mean pressures, and an also increased right ventricular work that was strongly correlated to the increased PAMP.

We have found a more homogeneous perfusion distribution with opened AVF than with closed one, along with higher PAP. Other conditions with an increased PAP generally show a more uniform distribution of pulmonary blood flow than conditions with normal levels of PAP. Then, as a result, a correspondingly closer to 1.0 ratio of upper to lower zone blood flow follows, as we also evidenced. The more uniform distribution of blood flow can be explained by the interaction among the pulmonary arterial, venous, and alveolar pressures. Dollery *et al*. studied fifteen patients with intracardiac shunts with and without pulmonary hypertension using radioactive carbon dioxide [24]. Whereas in five normal subjects the ratio of upper to lower zone clearance rates (blood flow) was 0.2, this was increased to 0.88 in six patients with pulmonary arterial hypertension. The patients with normal (or nearly normal) pulmonary artery pressures but increased pulmonary blood flow because of left-to-right intracardiac shunts also had a closer to 1.0 distribution of blood flow but with a smaller magnitude (0.8) than the patients with pulmonary arterial hypertension (0.88).

Taking in account that an AVF leads to a substantial increase in arterial and venous flow, conditions with increased pulmonary venous pressure are also worth discussing. With small increases in venous pressure, a more uniform distribution of blood flow is seen [25]. This can be partly explained on the basis of the three-zone-model [26] by the passive rise in pulmonary artery pressure which accompanies an increase in venous pressure. Since an AVF leads to a hyperkinetic circulation, it should be also remembered that moderate exercise sufficient to raise the mean pulmonary artery pressure by only 5 mm Hg causes a substantially more uniform distribution of pulmonary blood flow [25].

Real-time information on regional perfusion during mechanical ventilation, and its changes, may help elucidate the combined effects of mechanical ventilation settings and associated conditions such as AVF. One may question why, in these healthy lungs, the percentage of total pulmonary blood flow through the *Dorsal* ROI at AVF_closed_ (baseline condition) was 38.6±11.7 %. According to the investigational protocol, the mechanical ventilation settings were initially adjusted to maintain normoxia and normocapnia and remained unchanged during the whole experiment. Actually, both normoxia and normocapnia were sustained throughout the study. A recent experimental study measured pulmonary regional perfusion by EIT at 12 combinations of positive end-expiratory pressure (PEEP) and tidal volume (V_T_) in mechanically ventilated piglets with healthy and injured lungs [15]. In the healthy lungs under PEEP 5 cmH_2_O and V_T_ 7 ml/kg, the dorsal (dependent) lung region perfusion was around 30 %. In our animals, the measured levels of PEEP and V_T_ were 6.1±2.2 cmH_2_O and 6.5±1.2 ml/kg. We would like also to reinforce that we did the perfusion measurements during apnea and at end-expiration.

We have also found that the percentage of total pulmonary ventilation through the *Dorsal* ROI was 30.4±14.6. Anesthesia-induced atelectasis is a very well described phenomenon that occurs frequently in normal lungs under mechanical ventilation and general anesthesia [27–30]. The use or not of lung recruitment maneuvers as well as the applied PEEP level are some of the key factors involved. In particular, the application of a PEEP level of 10 cmH_2_O was shown to be needed to eliminate or reduce the occurrence of dorsal lung atelectasis during general anesthesia [29]. In our study no lung recruitment maneuvers were used, and the PEEP values were low. Noteworthy, the perfusion within atelectatic lung units is not mainly determined by mechanical factors. It has been demonstrated that hypoxic pulmonary vasoconstriction (HPV) [31] is the major (but not necessarily only) determinant of increased vascular resistance in an atelectatic lung region, and that passive mechanical factors do not measurably affect blood flow distribution within atelectatic lung units [32]. That fits also with observations by Benumof *et al*. [33], which corroborated that HPV is the main mechanism of reduced blood flow in atelectatic regions, not mechanical obstruction. It should also be taken in account that HPV is significantly stronger in pigs than in humans.

There are several limitations in the present study. First, we included a small number of animals, and this must thus be considered an exploratory and descriptive study. Second, we performed only a short-term study to describe acute changes. Thus, long-time adaptation mechanisms did not have time to develop. A third limitation comes from the fact that EIT-based regional perfusion analysis is something between a 2-D and 3-D one (although the finite mesh is 3-D, the electrodes were placed within a single plane). Although reasonably large, the effective thickness of the EIT cross-sectional slice (≈ 15 cm) varies with the size and shape of the animal [34], and one could never guarantee that most of the lung is being represented in all animals. Fourth, these data come from animals under mechanical ventilation and general anesthesia. It would be interesting to make a similar study in awake (spontaneous breathing) patients with AVF.

## Conclusions

We have used EIT for the first time for studying the acute effects of an arteriovenous fistula on regional distribution of pulmonary perfusion in a novel porcine model. In this new experimental model of hyperkinetic circulation caused by a large arteriovenous fistula, we documented an increased percentage of total pulmonary blood flow through the dorsal lung region as well as a more homogeneous perfusion distribution after reaching stable state.

## Figures and Tables

**Table 1:** Hemodynamic changes after arteriovenous fistula creation.

Parameter	AVF_closed_	AVF_open_	*P* value
*Qa (l/min)*	**_**	**2.1 ± 0.5**	**_**
*HR (/min)*	**95 ± 22**	**113 ± 26**	**0.001**
*ART(S) (mm Hg)*	**118 ± 15**	**107 ± 22**	**0.035**
*ART(D) (mm Hg)*	**79 ± 13**	**64 ± 15**	**0.002**
*ART(M) (mm Hg)*	**90 ± 14**	**77 ± 17**	**0.002**
*CVP (mm Hg)*	**4 ± 2**	**4 ± 2**	0.17
*PASP (mm Hg)*	**32 ± 10**	**38 ± 8**	**0.037**
*PAMP (mm Hg)*	**24 ± 7**	**29 ± 9**	**0.035**
*PCWP (mm Hg)*	**9 ± 4**	**7 ± 4**	0.32
*SvO* *_2_* * (%)*	**57.8 ± 10.4**	**64.5 ± 8.9**	**0.010**
*CO (l/min)*	**6.98 ± 2.48**	**9.15 ± 3.15**	**0.0003**
*% of CO through AVF*	**_**	**24 ± 4**	**_**
*SV (ml)*	**74 ± 20**	**82 ± 21**	0.08
*SVR (WU)*	**13.5 ± 4.4**	**11.2 ± 3.6**	**0.009**
*PVR (WU)*	**3.7 ± 1.5**	**3.4 ± 1.2**	0.28
*LV work (gm)*	**83.7 ± 31.2**	**80.3 ± 33.9**	0.58
*RV work (gm)*	**19.8 ± 6.9**	**27.2 ± 9.2**	**0.014**

Statistically significant results are in bold. Displayed values are averaged from all subjects. “AVF_closed_” data represent measurements obtained with closed arteriovenous fistula. “AVF_open_” data represent measurements obtained after 60 min of opened AVF after reaching stable state. Qa, arteriovenous fistula blood flow; HR, heart rate; ART, systemic arterial blood pressure; S, systolic; D, diastolic; M, mean; CVP, central venous pressure; PASP, pulmonary artery systolic pressure; PAMP, pulmonary artery mean pressure; PCWP, pulmonary capillary wedge pressure; SvO_2_, hemoglobin saturation in mixed venous blood; CO, cardiac output; SV, stroke volume; SVR, systemic vascular resistance; PVR, pulmonary vascular resistance; WU, Wood units; LV, left ventricle; RV, right ventricle.
